# 6-Bromo-1-ethyl-1*H*-2,1-benzothia­zin-4(3*H*)-one 2,2-dioxide

**DOI:** 10.1107/S1600536809002621

**Published:** 2009-01-28

**Authors:** Muhammad Shafiq, M. Nawaz Tahir, Islam Ullah Khan, Muhammad Nadeem Arshad, Muhammad Safdar

**Affiliations:** a Government College University, Department of Chemistry, Lahore, Pakistan; b University of Sargodha, Department of Physics, Sargodha, Pakistan; c University of Karachi, HEJ research Institute of Chemistry, Karachi, Pakistan

## Abstract

In the title compound, C_10_H_10_BrNO_3_S, the S atom is four-coordinated in a distorted tetra­hedral configuration with nearly equal S=O bond distances; the S—C and S—N bond lengths are 1.755 (3) and 1.649 (3) Å, respectively. The heterocyclic thia­zine ring adopts a twist conformation. Adjacent mol­ecules are attached to each other through inter­molecular C—H⋯O hydrogen bonds, forming *R*_2_^2^(8) and *R*_2_^2^(14) ring motifs. The mol­ecules are stabilized by intra- and inter­molecular hydrogen bonds, forming a three-dimensional polymeric network.

## Related literature

For previous work on benzothia­zines, see: Arshad *et al.* (2008 ▶); Shafiq, Khan *et al.* (2008 ▶); Shafiq, Tahir *et al.* (2008 ▶); Tahir *et al.* (2008 ▶). For puckering parameters, see: Cremer & Pople (1975 ▶). For graph-set motifs, see: Bernstein *et al.* (1995 ▶). For synthesis, see: Lombardino (1972 ▶).
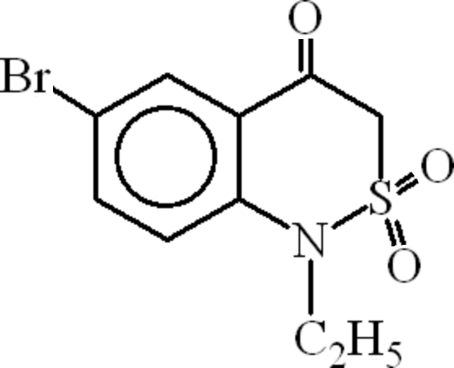


## Experimental

### 

#### Crystal data

C_10_H_10_BrNO_3_S
*M* *_r_* = 304.16
Triclinic, 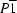

*a* = 7.7164 (2) Å
*b* = 7.9729 (3) Å
*c* = 10.4579 (3) Å
α = 86.767 (2)°
β = 75.773 (1)°
γ = 66.912 (2)°
*V* = 573.13 (3) Å^3^
*Z* = 2
Mo *K*α radiation
μ = 3.76 mm^−1^
*T* = 296 (2) K
0.28 × 0.16 × 0.12 mm

#### Data collection

Bruker Kappa APEXII CCD diffractometer
Absorption correction: multi-scan (*SADABS*; Bruker, 2005 ▶) *T*_min_ = 0.486, *T*_max_ = 0.639
12369 measured reflections
2846 independent reflections
1840 reflections with *I* > 2σ(*I*)
*R*_int_ = 0.034

#### Refinement

*R*[*F* ^2^ > 2σ(*F* ^2^)] = 0.038
*wR*(*F* ^2^) = 0.095
*S* = 1.02
2846 reflections
158 parameters
H atoms treated by a mixture of independent and constrained refinement
Δρ_max_ = 0.91 e Å^−3^
Δρ_min_ = −0.58 e Å^−3^

### 

Data collection: *APEX2* (Bruker, 2007 ▶); cell refinement: *APEX2*; data reduction: *SAINT* (Bruker, 2007 ▶); program(s) used to solve structure: *SHELXS97* (Sheldrick, 2008 ▶); program(s) used to refine structure: *SHELXL97* (Sheldrick, 2008 ▶); molecular graphics: *ORTEP-3 for Windows* (Farrugia, 1997 ▶) and *PLATON* (Spek, 2003 ▶); software used to prepare material for publication: *WinGX* (Farrugia, 1999 ▶) and *PLATON*.

## Supplementary Material

Crystal structure: contains datablocks global, I. DOI: 10.1107/S1600536809002621/at2711sup1.cif

Structure factors: contains datablocks I. DOI: 10.1107/S1600536809002621/at2711Isup2.hkl

Additional supplementary materials:  crystallographic information; 3D view; checkCIF report

## Figures and Tables

**Table 1:** Hydrogen-bond geometry (Å, °)

*D*—H⋯*A*	*D*—H	H⋯*A*	*D*⋯*A*	*D*—H⋯*A*
C2—H2⋯O1^i^	0.9300	2.4200	3.324 (4)	165.00
C3—H3⋯O2^ii^	0.9300	2.5900	3.418 (4)	148.00
C8—H8*A*⋯O3^iii^	0.89 (4)	2.57 (4)	3.252 (4)	134 (3)
C9—H9*B*⋯O3	0.93 (3)	2.29 (3)	2.850 (4)	118 (2)

Symmetry codes: (i) 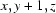; (ii) 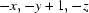; (iii) 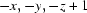.

**Fig. 1.**
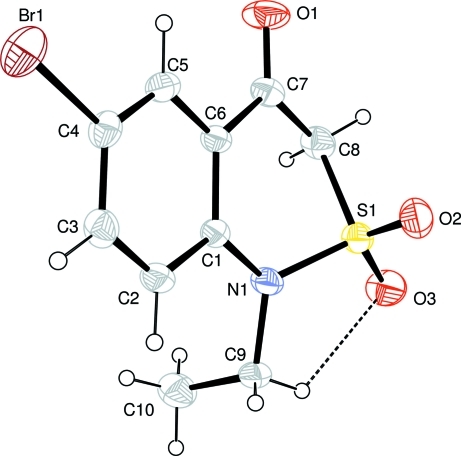
ORTEP drawing of the title compound, with the atom numbering scheme. The thermal ellipsoids are drawn at the 50% probability level. H atoms are shown by small circles of arbitrary radii. The dotted line shows the intramolecular H-bond.

**Fig. 2.**
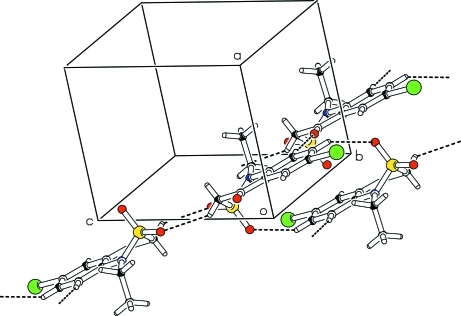
The partial packing figure (PLATON: Spek, 2003) which shows that intermolecular H-bonds form the ring motifs.

## supplementary crystallographic information

### Comment

Our group is involved in synthesizing various derivatives of benzothiazine molecule (Shafiq, Khan *et al.*, 2008; Shafiq, Tahir *et al.*, 2008; Tahir *et al.*, 2008; Arshad *et al.*, 2008) and their characterization by X-ray studies. We, here, report the title compound (I), (Fig 1), in this context.

In the title compound, the bromo-substituated benzene ring A (C1–C6), is almost planar with alternate distortions at individual C atoms. The Br atom is at a distance of 0.073 (4) Å from the r.m.s. plane of ring A. The thiazine ring B (S1/N1/C1/C6–C8) is in the twisted form. The maximum puckering (Cremer & Pople, 1975) amplitude, Q_T_, of ring A and ring B is 0.674 (2) Å. There exist an intramolecular H-bond of C—H···O type between the methylene group and the SO_2_ moiety. The intermolecular H-bonds [C8—H8A···.O3] and [C3—H3···.O2] (Table 1), joint the adjacent molecules forming ring motifs, (Bernstein *et al.*, 1995), *R*_2_^2^(8) and *R*_2_^2^(14), respectively. The three asymmetric units joint in this way, are further linked through [C2—H2···O1] H-bonds with carbonyl moiety (Fig 2).

### Experimental

The title compound was prepared in a three step scheme following the reported procedure (Lombardino, 1972). Starting material used was methyl-2-amino-5-bromo benzoate. It was reacted with methane sulfonyl chloride taking equimolar quantities, in dichloromethane. The pH was kept alkaline with triethylamine. The product of this step was then *N*-ethylated (ethyl iodide) and cyclized as reported in the above mentioned reference, to get the title compound which was recrystallized in ethanol for X-ray diffraction studies.

### Refinement

The H atoms of methylene group were located from a difference Fourier map and refined freely. H atoms were positioned geometrically, with C—H = 0.93 and 0.96 Å for aromatic and methyl H, and constrained to ride on their parent atoms, with U_iso_(H) = xU_eq_(C), where x = 1.5 for methyl H, and x = 1.2 for all other H atoms.

### Crystal data

**Table:**

C_10_H_10_BrNO_3_S	*Z* = 2
*M**_r_* = 304.16	*F*(000) = 304
Triclinic, *P*1	*D*_x_ = 1.762 Mg m^−^^3^
Hall symbol: -P 1	Mo *K*α radiation, λ = 0.71073 Å
*a* = 7.7164 (2) Å	Cell parameters from 2847 reflections
*b* = 7.9729 (3) Å	θ = 2.0–28.3°
*c* = 10.4579 (3) Å	µ = 3.76 mm^−^^1^
α = 86.767 (2)°	*T* = 296 K
β = 75.773 (1)°	Needle, colourless
γ = 66.912 (2)°	0.28 × 0.16 × 0.12 mm
*V* = 573.13 (3) Å^3^	

### Data collection

**Table:**

Bruker Kappa APEXII CCD diffractometer	2846 independent reflections
Radiation source: fine-focus sealed tube	1840 reflections with *I* > 2σ(*I*)
graphite	*R*_int_ = 0.034
Detector resolution: 7.40 pixels mm^-1^	θ_max_ = 28.3°, θ_min_ = 2.0°
ω scans	*h* = −10→9
Absorption correction: multi-scan (*SADABS*; Bruker, 2005)	*k* = −10→10
*T*_min_ = 0.486, *T*_max_ = 0.639	*l* = −13→13
12369 measured reflections	

### Refinement

**Table:**

Refinement on *F*^2^	Primary atom site location: structure-invariant direct methods
Least-squares matrix: full	Secondary atom site location: difference Fourier map
*R*[*F*^2^ > 2σ(*F*^2^)] = 0.038	Hydrogen site location: inferred from neighbouring sites
*wR*(*F*^2^) = 0.095	H atoms treated by a mixture of independent and constrained refinement
*S* = 1.02	*w* = 1/[σ^2^(*F*_o_^2^) + (0.0388*P*)^2^ + 0.3304*P*] where *P* = (*F*_o_^2^ + 2*F*_c_^2^)/3
2846 reflections	(Δ/σ)_max_ = 0.001
158 parameters	Δρ_max_ = 0.91 e Å^−^^3^
0 restraints	Δρ_min_ = −0.57 e Å^−^^3^

### Special details

**Table:**

Geometry. Bond distances, angles *etc*. have been calculated using the rounded fractional coordinates. All su's are estimated from the variances of the (full) variance-covariance matrix. The cell e.s.d.'s are taken into account in the estimation of distances, angles and torsion angles
Refinement. Refinement of *F*^2^ against ALL reflections. The weighted *R*-factor *wR* and goodness of fit *S* are based on *F*^2^, conventional *R*-factors *R* are based on *F*, with *F* set to zero for negative *F*^2^. The threshold expression of *F*^2^ > σ(*F*^2^) is used only for calculating *R*-factors(gt) *etc*. and is not relevant to the choice of reflections for refinement. *R*-factors based on *F*^2^ are statistically about twice as large as those based on *F*, and *R*- factors based on ALL data will be even larger.

### Fractional atomic coordinates and isotropic or equivalent isotropic displacement parameters (Å^2^)

**Table:**

	*x*	*y*	*z*	*U*_iso_*/*U*_eq_	
Br1	0.36240 (6)	0.15247 (6)	−0.35948 (3)	0.0686 (2)	
S1	−0.00069 (12)	0.19519 (10)	0.32296 (7)	0.0435 (3)	
O1	0.2170 (4)	−0.2056 (3)	0.0770 (3)	0.0705 (10)	
O2	−0.1737 (3)	0.2464 (3)	0.2787 (2)	0.0578 (8)	
O3	−0.0156 (4)	0.2267 (3)	0.4583 (2)	0.0668 (10)	
N1	0.1437 (4)	0.2891 (3)	0.2351 (2)	0.0450 (9)	
C1	0.1999 (4)	0.2535 (4)	0.0977 (3)	0.0358 (9)	
C2	0.2376 (5)	0.3826 (4)	0.0126 (3)	0.0444 (10)	
C3	0.2876 (5)	0.3511 (4)	−0.1214 (3)	0.0471 (11)	
C4	0.3017 (4)	0.1895 (4)	−0.1739 (3)	0.0423 (10)	
C5	0.2710 (4)	0.0580 (4)	−0.0934 (3)	0.0416 (10)	
C6	0.2210 (4)	0.0869 (4)	0.0432 (3)	0.0361 (9)	
C7	0.1935 (4)	−0.0623 (4)	0.1248 (3)	0.0427 (10)	
C8	0.1398 (6)	−0.0343 (4)	0.2718 (3)	0.0493 (11)	
C9	0.1564 (5)	0.4465 (4)	0.2950 (3)	0.0465 (11)	
C10	0.3622 (5)	0.4137 (5)	0.2925 (4)	0.0635 (14)	
H2	0.22872	0.49140	0.04727	0.0533*	
H3	0.31214	0.43846	−0.17724	0.0564*	
H5	0.28342	−0.05116	−0.12983	0.0499*	
H8A	0.065 (5)	−0.092 (5)	0.313 (4)	0.0591*	
H8B	0.247 (5)	−0.063 (5)	0.306 (3)	0.0591*	
H9A	0.092 (5)	0.550 (5)	0.245 (3)	0.0558*	
H9B	0.085 (5)	0.457 (4)	0.382 (3)	0.0558*	
H10A	0.43344	0.41151	0.20285	0.0948*	
H10B	0.36403	0.50981	0.34278	0.0948*	
H10C	0.42109	0.29879	0.33010	0.0948*	

### Atomic displacement parameters (Å^2^)

**Table:**

	*U*^11^	*U*^22^	*U*^33^	*U*^12^	*U*^13^	*U*^23^
Br1	0.0779 (3)	0.0861 (3)	0.0329 (2)	−0.0249 (2)	−0.0092 (2)	0.0002 (2)
S1	0.0540 (5)	0.0443 (4)	0.0344 (4)	−0.0260 (4)	−0.0036 (3)	0.0030 (3)
O1	0.113 (2)	0.0401 (13)	0.0672 (17)	−0.0416 (15)	−0.0168 (15)	−0.0010 (12)
O2	0.0464 (14)	0.0583 (14)	0.0673 (16)	−0.0232 (12)	−0.0074 (12)	0.0063 (12)
O3	0.098 (2)	0.0734 (16)	0.0341 (13)	−0.0463 (15)	−0.0033 (12)	0.0009 (11)
N1	0.0626 (17)	0.0467 (14)	0.0332 (14)	−0.0347 (14)	−0.0012 (12)	−0.0045 (11)
C1	0.0391 (16)	0.0356 (15)	0.0340 (16)	−0.0178 (13)	−0.0056 (12)	−0.0001 (12)
C2	0.0552 (19)	0.0351 (15)	0.0453 (19)	−0.0238 (15)	−0.0057 (15)	0.0010 (13)
C3	0.051 (2)	0.0462 (18)	0.0429 (19)	−0.0212 (16)	−0.0075 (15)	0.0110 (15)
C4	0.0425 (18)	0.0510 (18)	0.0307 (16)	−0.0164 (15)	−0.0074 (13)	0.0015 (14)
C5	0.0439 (18)	0.0377 (16)	0.0418 (18)	−0.0139 (14)	−0.0098 (14)	−0.0056 (14)
C6	0.0419 (17)	0.0324 (15)	0.0369 (16)	−0.0182 (13)	−0.0082 (13)	0.0005 (12)
C7	0.0487 (19)	0.0354 (16)	0.0491 (19)	−0.0198 (14)	−0.0158 (15)	0.0050 (14)
C8	0.061 (2)	0.0436 (18)	0.047 (2)	−0.0254 (18)	−0.0137 (17)	0.0121 (15)
C9	0.056 (2)	0.0423 (17)	0.0440 (19)	−0.0255 (17)	−0.0033 (16)	−0.0115 (15)
C10	0.065 (2)	0.066 (2)	0.071 (3)	−0.033 (2)	−0.023 (2)	−0.004 (2)

### Geometric parameters (Å, °)

**Table:**

Br1—C4	1.893 (3)	C6—C7	1.477 (4)
S1—O2	1.421 (3)	C7—C8	1.496 (4)
S1—O3	1.420 (2)	C9—C10	1.499 (6)
S1—N1	1.649 (3)	C2—H2	0.9300
S1—C8	1.755 (3)	C3—H3	0.9300
O1—C7	1.202 (4)	C5—H5	0.9300
N1—C1	1.406 (4)	C8—H8A	0.89 (4)
N1—C9	1.481 (4)	C8—H8B	0.92 (4)
C1—C2	1.393 (4)	C9—H9A	0.98 (4)
C1—C6	1.409 (4)	C9—H9B	0.93 (3)
C2—C3	1.369 (4)	C10—H10A	0.9600
C3—C4	1.383 (4)	C10—H10B	0.9600
C4—C5	1.364 (4)	C10—H10C	0.9600
C5—C6	1.393 (4)		
			
Br1···Br1^i^	3.5704 (5)	C2···H9A	2.61 (3)
Br1···H8B^ii^	3.00 (4)	C2···H10A	2.8500
O1···C2^iii^	3.324 (4)	C9···H2	2.5500
O2···C6	3.254 (4)	C10···H2	2.9400
O2···C10^iv^	3.263 (5)	H2···O1^ix^	2.4200
O2···C3^v^	3.418 (4)	H2···C9	2.5500
O3···C8^vi^	3.252 (4)	H2···C10	2.9400
O1···H9A^iii^	2.84 (4)	H2···H9A	2.0600
O1···H2^iii^	2.4200	H2···H10A	2.4300
O1···H5	2.4700	H3···O2^v^	2.5900
O2···H10C^iv^	2.9000	H5···O1	2.4700
O2···H5^vii^	2.7400	H5···O2^vii^	2.7400
O2···H3^v^	2.5900	H8A···O3^vi^	2.57 (4)
O3···H9B	2.29 (3)	H8B···Br1^ii^	3.00 (4)
O3···H8A^vi^	2.57 (4)	H9A···O1^ix^	2.84 (4)
O3···H9B^viii^	2.90 (3)	H9A···C2	2.61 (3)
C2···O1^ix^	3.324 (4)	H9A···H2	2.0600
C2···C10	3.348 (5)	H9B···O3	2.29 (3)
C2···C2^v^	3.472 (5)	H9B···O3^viii^	2.90 (3)
C3···O2^v^	3.418 (4)	H9B···H9B^viii^	2.49 (4)
C6···O2	3.254 (4)	H10A···C1	2.9900
C8···O3^vi^	3.252 (4)	H10A···C2	2.8500
C10···C2	3.348 (5)	H10A···H2	2.4300
C10···O2^x^	3.263 (5)	H10C···O2^x^	2.9000
C1···H10A	2.9900		
			
O2—S1—O3	118.87 (16)	S1—C8—C7	112.1 (2)
O2—S1—N1	110.91 (14)	N1—C9—C10	111.6 (3)
O2—S1—C8	106.97 (18)	C1—C2—H2	120.00
O3—S1—N1	107.50 (16)	C3—C2—H2	120.00
O3—S1—C8	111.51 (15)	C2—C3—H3	120.00
N1—S1—C8	99.36 (16)	C4—C3—H3	120.00
S1—N1—C1	117.1 (2)	C4—C5—H5	120.00
S1—N1—C9	118.39 (19)	C6—C5—H5	120.00
C1—N1—C9	121.6 (2)	S1—C8—H8A	104 (2)
N1—C1—C2	120.3 (3)	S1—C8—H8B	104 (2)
N1—C1—C6	121.0 (3)	C7—C8—H8A	113 (3)
C2—C1—C6	118.7 (3)	C7—C8—H8B	112 (2)
C1—C2—C3	120.7 (3)	H8A—C8—H8B	111 (3)
C2—C3—C4	120.1 (3)	N1—C9—H9A	104 (2)
Br1—C4—C3	119.3 (2)	N1—C9—H9B	105 (2)
Br1—C4—C5	120.0 (2)	C10—C9—H9A	115 (2)
C3—C4—C5	120.7 (3)	C10—C9—H9B	110 (3)
C4—C5—C6	120.1 (3)	H9A—C9—H9B	111 (3)
C1—C6—C5	119.6 (3)	C9—C10—H10A	109.00
C1—C6—C7	122.9 (3)	C9—C10—H10B	110.00
C5—C6—C7	117.4 (3)	C9—C10—H10C	109.00
O1—C7—C6	122.2 (3)	H10A—C10—H10B	109.00
O1—C7—C8	119.4 (3)	H10A—C10—H10C	109.00
C6—C7—C8	118.4 (3)	H10B—C10—H10C	109.00
			
O2—S1—N1—C1	−56.8 (3)	N1—C1—C6—C5	178.0 (3)
O2—S1—N1—C9	104.3 (2)	N1—C1—C6—C7	−2.4 (5)
O3—S1—N1—C1	171.7 (2)	C2—C1—C6—C5	−2.6 (5)
O3—S1—N1—C9	−27.2 (3)	C2—C1—C6—C7	177.1 (3)
C8—S1—N1—C1	55.5 (3)	C1—C2—C3—C4	−0.2 (6)
C8—S1—N1—C9	−143.4 (3)	C2—C3—C4—Br1	177.9 (3)
O2—S1—C8—C7	61.0 (3)	C2—C3—C4—C5	−1.7 (6)
O3—S1—C8—C7	−167.5 (3)	Br1—C4—C5—C6	−178.2 (3)
N1—S1—C8—C7	−54.3 (3)	C3—C4—C5—C6	1.3 (5)
S1—N1—C1—C2	149.4 (3)	C4—C5—C6—C1	0.8 (5)
S1—N1—C1—C6	−31.2 (4)	C4—C5—C6—C7	−178.8 (3)
C9—N1—C1—C2	−11.1 (5)	C1—C6—C7—O1	−178.0 (3)
C9—N1—C1—C6	168.3 (3)	C1—C6—C7—C8	0.4 (5)
S1—N1—C9—C10	125.2 (3)	C5—C6—C7—O1	1.7 (5)
C1—N1—C9—C10	−74.6 (4)	C5—C6—C7—C8	180.0 (3)
N1—C1—C2—C3	−178.3 (3)	O1—C7—C8—S1	−150.2 (3)
C6—C1—C2—C3	2.2 (5)	C6—C7—C8—S1	31.4 (5)

Symmetry codes: (i) −*x*+1, −*y*, −*z*−1; (ii) −*x*+1, −*y*, −*z*; (iii) *x*, *y*−1, *z*; (iv) *x*−1, *y*, *z*; (v) −*x*, −*y*+1, −*z*; (vi) −*x*, −*y*, −*z*+1; (vii) −*x*, −*y*, −*z*; (viii) −*x*, −*y*+1, −*z*+1; (ix) *x*, *y*+1, *z*; (x) *x*+1, *y*, *z*.

### Hydrogen-bond geometry (Å, °)

**Table:**

*D*—H···*A*	*D*—H	H···*A*	*D*···*A*	*D*—H···*A*
C2—H2···O1^ix^	0.9300	2.4200	3.324 (4)	165.00
C3—H3···O2^v^	0.9300	2.5900	3.418 (4)	148.00
C8—H8A···O3^vi^	0.89 (4)	2.57 (4)	3.252 (4)	134 (3)
C9—H9B···O3	0.93 (3)	2.29 (3)	2.850 (4)	118 (2)

Symmetry codes: (ix) *x*, *y*+1, *z*; (v) −*x*, −*y*+1, −*z*; (vi) −*x*, −*y*, −*z*+1.
